# Targeted high throughput sequencing in clinical cancer Settings: formaldehyde fixed-paraffin embedded (FFPE) tumor tissues, input amount and tumor heterogeneity

**DOI:** 10.1186/1755-8794-4-68

**Published:** 2011-09-29

**Authors:** Martin Kerick, Melanie Isau, Bernd Timmermann, Holger Sültmann, Ralf Herwig, Sylvia Krobitsch, Georg Schaefer, Irmgard Verdorfer, Georg Bartsch, Helmut Klocker, Hans Lehrach, Michal R Schweiger

**Affiliations:** 1 Max Planck Institute for Molecular Genetics, Ihnestr. 63-73, 14195 Berlin, Germany; 2 Free University Berlin, Department of Biology, Chemistry and Pharmacy, Takustrasse 3, 14195 Berlin, Germany; 3 Unit Cancer Genome Research, DKFZ German Cancer Research Center and National Center for Tumor Diseases, Im Neuenheimer Feld 460, 69120 Heidelberg, Germany; 4 Innsbruck Medical University, Department of Urology, Anichstr. 35, A 6020 Innsbruck, Austria; 5 Innsbruck Medical University, Department of Pathology, Muellerstr. 40, A-6020 Innsbruck, Austria; 6 Innsbruck Medical University, Department for Medical Genetics, Molecular and Clinical Pharmacology, Division of Human Genetics, Schöpfstraße 4, 6020 Innsbruck, Austria

## Abstract

**Background:**

Massively parallel sequencing technologies have brought an enormous increase in sequencing throughput. However, these technologies need to be further improved with regard to reproducibility and applicability to clinical samples and settings.

**Methods:**

Using identification of genetic variations in prostate cancer as an example we address three crucial challenges in the field of targeted re-sequencing: Small nucleotide variation (SNV) detection in samples of formalin-fixed paraffin embedded (FFPE) tissue material, minimal amount of input sample and sampling in view of tissue heterogeneity.

**Results:**

We show that FFPE tissue material can supplement for fresh frozen tissues for the detection of SNVs and that solution-based enrichment experiments can be accomplished with small amounts of DNA with only minimal effects on enrichment uniformity and data variance.

Finally, we address the question whether the heterogeneity of a tumor is reflected by different genetic alterations, e.g. different foci of a tumor display different genomic patterns. We show that the tumor heterogeneity plays an important role for the detection of copy number variations.

**Conclusions:**

The application of high throughput sequencing technologies in cancer genomics opens up a new dimension for the identification of disease mechanisms. In particular the ability to use small amounts of FFPE samples available from surgical tumor resections and histopathological examinations facilitates the collection of precious tissue materials. However, care needs to be taken in regard to the locations of the biopsies, which can have an influence on the prediction of copy number variations. Bearing these technological challenges in mind will significantly improve many large-scale sequencing studies and will - in the long term - result in a more reliable prediction of individual cancer therapies.

## Background

According to the world health organization (WHO) malignant neoplasms are the most common cause of death worldwide in 2010 [1]. We now know that human solid tumors, which account for the majority of all human cancers, result from the accumulation of numerous genetic and epigenetic alterations that finally lead to the deregulation of protein-encoding genes [2-10].

Previous efforts to identify protein-encoding cancer genes were limited by insufficient technologies to detect genomic alterations on a global scale. Over the last years more advanced technologies such as next generation sequencing (NGS) technologies have been developed to detect the various patterns of mutations and rearrangements in individual cancer genomes revealing the complexity of tumor genetics [11]. These NGS technologies promise to bring about a revolution in cancer genomics such that it becomes feasible to describe the complex genetic networks underlying tumors and thus to identify pathomechanisms of tumor progression and therapy resistance [12-16].

In this regard first whole genome sequences have been published. For example, sequencing of a cytogenetically normal acute myeloid leukemia genome has revealed eight somatic mutations [14]. Within a similar range is the profile of a sequenced breast tumor with 32 non-synonymous somatic mutations [15]. Recently the complete genomes of lung cancer and melanoma cell lines have been analyzed and indicate correlations between DNA repair mechanisms and mutational spectra [17,18].

However, even though the power of next generation sequencing (NGS) technologies is enormous, remarkably few studies on cancer genomes have been published so far. This is mainly due to the fact that NGS is still relatively cost - and time - intensive and that bioinformatics analyses of tumor tissues are not only challenging, but also need a lot of time - this is likely to be the major bottleneck in the future. One solution to these drawbacks is to increase the sequencing output by focusing on coding DNA regions [11,19,20]. Several targeted DNA enrichment technologies to reduce sequence complexity are available [21-27]. These technologies have been mainly developed using large amounts of input DNA generated from blood samples. To identify somatic mutations in solid tumors, DNA has to be extracted from tissues; with often limited access and amounts of extracted DNA. Formalin fixed and paraffin embedded (FFPE) tissue samples, which are archived on a routine basis in pathology departments, could render more and rare conditions accessible. Although FFPE tissue was successfully used for low-coverage whole genome sequencing and copy number detection it is not known if it can be taken for SNV and InDel detection after targeted enrichment strategies [28].

Here, we have specifically addressed cancer-relevant technical questions for targeted sequencing in cancer genomics. We investigated whether FFPE tissue material can be used for targeted re-sequencing applications. We further evaluated the reproducibility and uniformity of the experiments and the effect of modifications such as DNA input amounts. Finally we addressed the question whether the heterogeneity of the tumor as seen by a pathologist is reflected by different mutation patterns or copy number alterations, e.g. if the localization of the biopsy matters. For this, we established quality standards for targeted re-sequencing experiments which can be also used for round-robin tests in clinical diagnostics.

## Methods

### Prostate tissue collection and preparation

Frozen and paraffin-embedded prostate tissue samples were obtained from five patients who had undergone radical prostatectomy at the Department of Urology, Innsbruck Medical University [29,30]. Immediately after surgery, the prostate specimens were cooled and sent to the pathologist, who performed a rapid section and isolated a prostate slice that was embedded in Tissue-Tek OCT Compound (Sakura Finetek, Staufen, Germany), snap-frozen in liquid nitrogen, and stored at -80°C until use. Pathological and clinical data were retrieved from the clinical databases and patients health records. The study was approved by the ethics committee at the Innsbruck Medical University and is in compliance with the Helsinki Declaration (UN3174 and AM3174).

### Isolation of DNA samples from prostate tissues

DNA samples were isolated from radical prostatectomy specimens of five patients. For isolation of DNA, 3 μm sections of the frozen specimens were prepared and stained with hematoxylin and eosin for pathological analysis and exact localization of the tumors. For each tumor sample, a paired benign (histopathologically normal) counterpart region distant from the tumor focus was identified. Selection of different foci was based on differences of histological and morphological phenotypes and was performed and controlled on the basis of HE stainings and P63/AMACR double immunostainings. P63 as a basal epithelial cell marker is absent in tumors, and tumor cells are positive for AMACR. In each case the two markers displayed different histopathological gradings, in two cases Gleason patterns 3+4 in the low grade focus and 4+5 in the high grade focus, the third case displayed an additional tertiary pattern 5 in the high grade focus. Subsequently, depending on the tumor area, 5-10 consecutive 10 μm sections were cut and carefully macro-dissected for isolation of tumor and benign regions, and the tissue pieces were collected in pre-cooled DNase/RNase free 1.7 ml micro-centrifuge tubes (Costar, Corning, MA, USA). The number of consecutive slides used for macrodissection was adjusted in each case to approximately 5-10 cm^2 ^of overall tissue section, which corresponds to approximately 5-10 mg of tissue and yielded between 2 and 9 μg of DNA. For DNA isolation, the EZ1 DNA tissue kit (Qiagen, Hilden, Germany) was used and the isolation was performed according to the protocol recommended by the supplier on a BioRobot EZ1 (Qiagen) equipped with the EZ1 tissue card. To increase DNA yield, the solubilization buffer was supplemented with additional 40 μl of Proteinase K solution (Roche, Basel Switzerland) and protease digestion was carried out over night at 56°C with repeatedly mixing during the first hours of incubation. After sample isolation the DNA amount was determined by UV spectroscopy using a Nanodrop instrument (PEQLAB Biotechnology, Erlangen Germany) and the quality was assessed by calculating the A260/280 ratio, which had to be ≥1.8.

For isolation of DNA from paraffin-embedded tissue specimens the EZ1 DNA tissue kit (Qiagen) procedure was slightly modified. Combined sections of each sample were suspended in 200 μl of sample extraction buffer G2. Samples were incubated for 5 min at 75°C with vigorous mixing (1400 rpm) on a thermomixer (Eppendorf). Thereafter the incubation temperature was lowered to 56°C and 10 μl of protease K solution (Roche) were added. Incubation at 56°C with continuous shaking was continued for an hour. During that hour samples were suspended 2-3 times by pipetting up and down several times to facilitate dissolution of the tissue samples. Afterwards additional 40 μl of protease K solution were added and the incubation at 56°C was continued over night. On the next morning additional 20 μl of protease solution was added and the incubation with shaking continued for 1 hour. Then the samples were centrifuged in a table centrifuge (Eppendorf) at 10000 g for 1 min to pellet all insoluble material and the supernatant was transferred to a fresh 2 ml sample tube.

DNA was isolated with an EZ1 BioRobot (Qiagen) equipped with an EZ1 DNA Paraffin Section Card using the EZ1 DNA Tissue Kit according to the instructions for this instrument. At the end of the purification procedure the DNA was eluted in 50 μl of RNAse/DNAse free water and the DNA concentration was measured using a nanodrop photometer (Peqlab, Erlangen Germany).

### DNA capturing of selected regions (3.9 Mb and 52 Mb)

The library preparation was performed according to Agilent's SureSelect protocol for Illumina single end sequencing with slight modifications. In brief, 0.5-3.0 μg of genomic DNA was sheared for 90 sec on a Covaris™ instrument set (duty cycle 20%, intensity 5 and 200 cycles per burst). The fragmented DNA (200-300 bp) was re-quantified with the Agilent 2100 Bioanalyzer 7500 chip. The following end repair reaction was performed to generate blunt-end fragments with 5'-phosphorylated ends. For the adapter ligation the "A" bases were added to the 3'-end of the DNA fragment. The adapters (5'GATCGGAAGAGCTCGTATGCCGTCTTCTGCTTG3') and (5'ACACTCTTTCCCTA-CACGACGCTCTTCCGATCT3') were used in a 10:1 molar ratio to raw genomic DNA.

The ligation products were purified and size selected with a range of 200-350 bp by agarose gel electrophoresis at 120 V for 1 h. The amplification of the library was performed with the Phusion High-Fidelity PCR master mix with HF buffer (Finnzymes) using Illumina PCR primers 1.1

(5'AATGATACGGCGACCACCGAGATCTACACTCTTTCCCTACACGACGCTTCCGATCT3') and 2.1 (5'CAA-GCAGAAGACGGCATACGAGCTCTTCCGATCT3') for 14 cycles.

For the hybrid selection the libraries were adjusted to 500 ng in 3.4 μl H_2_O and added to the SureSelect Block solutions. This mixture was heated at 95°C for 5 min and held for 5 min at 65°C. The library was then mixed with the prewarmed hybridization buffer (5 min at 65°C) and SureSelect oligo capture library mix (2 min at 65°C). After 24 h incubation at 65°C, the hybridization mix was added to 500 ng (50 μl) of M-280 streptavidin Dynabeads (Invitrogen), and the incubation was continued for 30 min at room temperature (RT). The beads were pulled down and washed once at RT for 15 min with 500 μl of SureSelect wash buffer 1, followed by three 10 min washes at 65°C with 500 μl of prewarmed SureSelect wash buffer 2. Hybrid-selected DNA was eluted with 50 μl of Elution buffer and incubated for 10 min at RT. After the pull down of the beads, the supernatant was transferred to a tube containing 50 μl of Neutralization buffer and the samples were desalted and concentrated on a QIAquick MinElute column and subsequently eluted in 30 μl Elution buffer. The post amplification step was performed with the Herculase polymerase and the SureSelect GA PCR-Primer-mix for 14 cycles.

### Quality control and NGS Sequencing

Quantification of the SureSelect captured library: Before sequencing, the samples were re-quantified with two methods. First, the size and concentration was checked on the Agilent 2100 Bioanalyzer and in a second step the enrichment efficiency was estimated by qPCR (Applied Biosystems) using a primerset for an enriched exon (fw: ATCCCGGTTGTTCTTCTGTG and rv: TTCTGGCTCTGCTGTAGGAAG) and a primerset in an intron region as a negative control (fw: AGGTTTGCTGAGGAACCTTGA and rv: ACCGAAACATCCTGGCTACAG). In general the CT-values of target and control fragments differed by 6 to 10, thus confirming a very good enrichment of our target regions.

After diluting the captured libraries to 10 nM, Genome Analyzer single read flow cells were prepared on the supplied Illumina cluster station and 36 bp single end reads on the Illumina Genome Analyzer IIx platform were generated following the manufacturer's protocol. Images from the instrument were processed using the manufacturer's software to generate FASTQ sequence files.

### Affymetrix SNParray

Cryo-embedded tissue material was genotyped on the Affymetrix 6.0 array, according to the manufacturer's protocol. Array positions with a quality score (p-value) < 0.01 were used as a 'gold standard' for the comparison with the sequencing data. Sequencing data positions within the enriched regions were used if their coverage exceeded 3-fold. This generated 6, 127 and 6, 122 positions for cryo and FFPE tissue, respectively, that were eligible for comparison. To determine false positive and false negative rates, we set the array data as standard and distinguished between reference call and SNP call depending on the array data.

### Bioinformatics analyses

Alignment: Raw reads were mapped to the golden path version hg19 using the bwa 0.5.8 alignment tool with default parameters. Sequences were deposited at the European Genome-phenome Archive [EGA: EGAS00001000136]. Enrichment statistics were calculated for target regions extended by 100 bp on either side. A read had to have at least one base within the target region to be evaluated "on target".

Coverage uniformity: The coefficient of variation was calculated for normalized mean coverages per exon. Normalization was done by a fixed factor per tissue sample to adjust the median coverage over all exons to the same level across all samples. For each two way comparison per exon we plotted the mean coverage of the exon with lower coverage on the x-axis. To examine the GC content dependent coverage for FFPE preparations for all exons the GC content was counted and exons were combined according to their GC content in step sizes of 0.1%. The basewise average exon coverage was averaged within each bin.

Normalized coverage-distribution plots were calculated as follows: The mean coverage per exon was divided by the overall mean coverage of all exons as normalized coverage (x-axis). The fraction of bait-covered exons in the genome achieving coverages equal or lower than the overall mean coverage is indicated on the y-axis.

Sorted coverage plots: Exons were sorted by their mean coverage and plotted along the x-axis. Coverage was plotted on the y-axis using a log10 scale.

Variant detection and comparison: Initial SNV and InDel detection was done using samtools 0.1.8 for each sample separately. Detected SNVs were required to have a Phred-scaled SNV probability greater or equal 20 and the SNV had to be present in at least 15% of all reads at a given position. A two step procedure was then applied to call the SNVs for comparison. SNVs detected by our criteria in one preparation were then examined in the second preparation to see if the SNV was found in at least one read. Discordant positions were determined by complimentary comparisons: SNVs called in preparation A had not to be found in preparation B or vice versa. Divergent positions for the snap frozen versus FFPE comparison could be stratified into false positive and false negative, assuming the snap frozen preparation as reference. For somatic SNV detection from two biopsies of the same prostate cancer tumor the Phred-scale cutoff was required to be greater or equal to 20 and the SNV was required to be found in both tumor foci in at least 4 reads but not in the corresponding benign tissue with a minimal coverage of 10 fold.

### Determination of copy number variations

After the DNA fragments were mapped aligned DNA read frequencies were determined for chromosomal intervals (bins) of 55-190 Kb. Interval sizes were chosen individually for each chromosome so that a minimal count of 600 reads per bin was achieved to ensure even data variance across the genome. The log2 ratio of tumor versus benign counts per bin was calculated and normalized by setting the genome wide median of the ratios to zero. To visualize copy number changes we calculated a running median of 20 bins using the lowess function in R. Differences in copy number between the two foci of one tumor were visualized by calculating the difference of the two running median vectors. Differences greater or equal 0.2 were highlighted.

## Results

### FFPE tissue can be used for targeted DNA capturing experiments and SNV detection

Thousands of patient samples are stored in pathology departments as formalin fixed and paraffin embedded (FFPE) tissues and provide an excellent source for molecular genetic studies. Previously we have shown that whole genome sequencing can be performed with this material [28,31].

**Figure 1:**
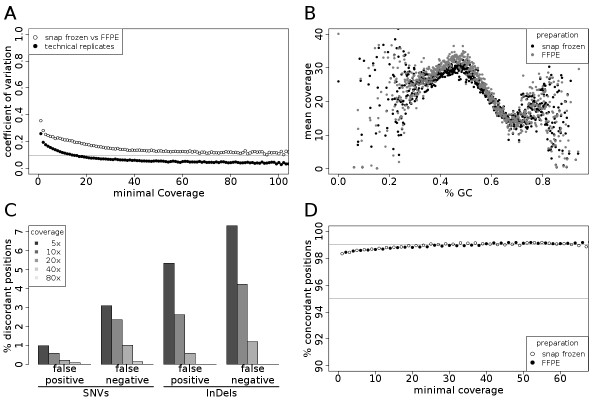
**Comparison of FFPE and snap frozen tissue material for whole exome re-sequencing approaches**. (**A**) Exonwise coverage comparison of snap frozen and FFPE DNA preparations for a benign tissue sample. Coefficients of variation are calculated exonwise and plotted by the smallest coverage of each exon-exon comparison. As reference, coefficients of variation were calculated for two sequencing replicates of the snap frozen preparation (**B**) Mean coverage by GC content for snap frozen and FFPE DNA preparations. All exons were split into 800 bins by GC content and the average exon coverage was averaged within each bin. (**C**) Comparison of SNVs and InDels detected in snap frozen and FFPE DNA preparations for a benign tissue sample. False negative SNVs/InDels are detected in the snap frozen preparation but not in the FFPE preparation. False positive SNVs/InDels are detected in the FFPE preparation but not in the snap frozen preparation. (**D**) Comparison of SNVs detected in snap frozen and FFPE DNA preparations with Affymetrix SNP array 6.0 plotted by minimal coverage.

Since many large-scale sequencing projects are now directed towards exome sequencing strategies, the question remains whether targeted re-sequencing on FFPE tissue might be possible. We therefore divided prostate tissue samples of radical prostatectomy specimens and stored one part as snap frozen tissue blocks, the other as FFPE material (Figure 1, Additional file 1, Methods). We used material from both preservation technologies for DNA extraction and subsequent hybridization for DNA capturing followed by Illumina sequencing of the "whole Exome" target region (52 Mb) as well as for a 3.9 Mb custom designed target region (Table 1 Additional file 2, Table S1). One caveat of next generation sequencing protocols from FFPE material is the high temperature (above 90°C) needed to melt the paraffin, which results in a significant fraction of single stranded DNA. However, for the subsequent library preparation step double stranded DNA is required. We found that an initial heating step to 75°C for 5 min is sufficient to melt the paraffin and preserve dsDNA. (Detailed protocols for the preparation of DNA, libraries and the capturing are provided in the Material and Method section.) For both DNA materials - snap frozen as well as FFPE stored - we identified an average of 75% of the sequencing reads located within the whole exome target region and more than 99% of the regions were captured by at least one read (Table 1, Additional file 2, Table S3). Both preservation technologies have similar coverage profiles and we found a high degree of correlation of enrichment per exon between experiments, which is depicted as coefficient of variation (Figure 1A, Additional file 1 Figure S1A, B). Even though the coefficient of variation is low within the same preparation technology, e.g. snap frozen versus snap frozen and FFPE versus FFPE, the variation is higher between snap frozen and FFPE. Thus, one should preferably remain within the same tissue preparation technology for one set of experiments (Additional file 1, Figure S1C). It is known that FFPE tissues are prone to spontaneous deamination of guanine and cytosine during the tissue preservation process and/or during storage and that the Illumina sequencing technology is biased for underrepresentation and reduced quality at loci with extreme base compositions [32]. In this regard, we found a slight, but not significant, shift in the GC-dependent coverage profile between the snap-frozen and FFPE tissue (Figure 1B, Additional file 1, Figure S1D).

**Table 1:** Sequencing statistics

ID	sample specification	Target size	# uniquely aligned reads	% reads on target	# of enriched regions	# of SNVs called (20×)
Pat1_B	Snap-frozen	52 Mb	66, 114, 467	75.4%	200, 175	18, 287
Pat1_B	FFPE	52 Mb	71, 590, 872	74.7%	200, 032	17, 810
Pat11_B	Snap-frozen	3.9 Mb	28, 043, 981	62.8%	12, 274	3, 000
Pat11_T	Snap-frozen	3.9 Mb	18, 302, 565	71.3%	12, 226	2, 511
Pat11_B	FFPE	3.9 Mb	9, 311, 629	67.1%	12, 175	2, 577
Pat11_T	FFPE	3.9 Mb	15, 928, 525	73.7%	12, 254	2, 883
						
Pat2_T	500 ng	3.9 Mb	8, 760, 773	81.1%	12, 220	2, 652
Pat2_T	1500 ng	3.9 Mb	9, 686, 320	79.9%	12, 243	2, 848
Pat2_T	3000 ng	3.9 Mb	6, 810, 410	80.4%	12, 204	2, 602
						
Pat3_B	Benign tissue	3.9 Mb	19, 617, 926	67.7%	12, 122	2, 002
Pat3_T	Focus-1	3.9 Mb	28, 798, 280	68.9%	12, 227	2, 328
Pat3_T	Focus-2	3.9 Mb	31, 939, 154	69.6%	11, 142	2, 513
Pat4_B	Benign tissue	3.9 Mb	8, 878, 742	66.2%	12, 281	2, 662
Pat4_T	Focus-1	3.9 Mb	8, 768, 332	64.2%	12, 288	2, 645
Pat4_T	Focus-2	3.9 Mb	9, 178, 790	65.3%	12, 269	2, 706
Pat5_B	Benign tissue	3.9 Mb	25, 957, 461	63.2%	12, 274	2, 646
Pat5_T	Focus-1	3.9 Mb	65, 372, 578	64.4%	12, 261	2, 388
Pat5_T	Focus-2	3.9 Mb	25, 957, 461	63.0%	11, 157	2, 342

ID: Identification number of the patient's tissue with B for benign tissue and T for tumor tissue.

To assess the effect of coverage depth on the sensitivity and specificity of sequence variant detection, we used genotype calls of an Affymetrix SNP array 6.0 from the cryo material and compared each position to the whole exome sequencing data. For both tissue preparations we achieved very similar accuracies above 98%, even at coverages down to 10× (Figure 1D).

Next we investigated the reproducibility of single nucleotide variation (SNV) detection in snap frozen versus FFPE tissues. We found 179 (1.2%) discordant loci investigating positions with at least 20-fold coverage. The potential artifacts can be grouped into false positives, e.g. a SNV is found in FFPE tissue without evidence in snap frozen tissue and false negative SNVs, where a SNV is found in snap frozen but not in FFPE material. Of the discordant loci we found 149 (0.99%) potential false positives with all but four that can be explained by processes likely to occur during formalin fixation, as e.g. deamination (C > T, A > G, 76 Loci, 53%). As false negative SNVs, namely SNVs found in snap frozen preparations but not FFPE preparations, we found 30 loci (0.2%) at a coverage level of greater than 20×. We next addressed the question if the differences detected can be overcome using more stringent coverage cutoffs (Figure 1C). While at 40× coverage 12 (0.19%) discordant loci were found, no discordance is left at 80× coverage. This also holds true for the custom designed sequencing of a 3.9 Mb region in tumor tissues (Additional file 1, Figure S1E).

In addition to SNVs we also detected insertions and deletions (InDels) and compared InDels detected in snap frozen versus FFPE tissues at a coverage cutoff of 20×. Discordant positions were found more frequent for InDels as opposed to SNVs with 8 (1.17%) loci as false positive and 4 (0.58%) loci as false negative positions. Again, higher coverage levels led to a lower percentage of discordant InDels, with no differences found at a coverage level of 40× (Figure 1C, Additional file 1, Figure 1F).

### Targeted sequence enrichment for small amounts of input DNA

**Figure 2:**
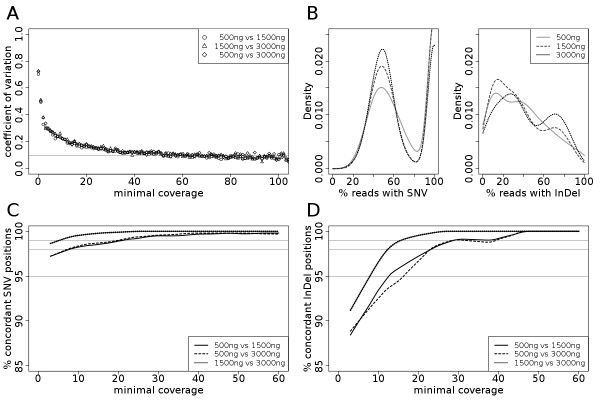
**Different DNA amounts for targeted re-sequencing approaches**. (**A**) Exonwise coverage comparisons obtained with different amounts of input DNA. Coefficient of variations were calculated for each comparison and plotted by the smallest coverage of each exon-exon comparison. (**B**) Variant/Reference ratio distribution for different amounts of input DNA. Depicted is the density curve for each preparation and distribution. (**C**) Comparison of SNVs detected with different amounts of input DNA. The Y-axis depicts the percentage of foci in concordance for different preparations at different coverage levels. (**D**) Comparison of InDels detected with different amounts of input DNA. The y-axis depicts the percentage of foci in concordance for different preparations at different coverage levels.

An important objective of technology development is to lower the amount of input DNA required. To this end, we used the targeted enrichment of 3.9 Mb distributed over 12, 366 independent regions and performed enrichment experiments with three different amounts of DNA (500 ng, 1500 ng, 3000 ng) obtained from frozen prostate cancer tissues. We found the enrichment efficiency to be similar for all three DNA amounts: Approximately 80% of sequencing reads mapped to the target regions and more than 98% of targets were hit at least once (Table 1, Additional file 2, Table S3). All three preparations had very similar global coverage profiles. In addition we found a high degree of correlation of enrichment per exon between experiments with a coefficient of variation lower than 0.2 at 20× coverage for all comparisons made (Figure 2A, Additional file 1, Figure S2). The enrichment is highly uniform and reproducible across several experiments (Additional file 1, Figure S2, S3).

Since smaller amounts of DNA might lead to a decreased sample complexity, and thereby to increased data variance, we calculated and visualized the variant/reference ratio distributions for different DNA amounts at a coverage level of 50× for SNVs and InDels (Figure 2B). In an ideal situation a heterozygous position would have 50% reads showing the variant - a variant/reference ratio of 0.5. Based on the ratios we find a slightly broader distribution for small amounts of input DNA which is also shifted towards higher ratios. The slightly lowered complexity of the samples with decreased DNA input amounts is also reflected in the number of unique start sites: For 500 ng input material we received 40% of the expected unique start sites (calculated in relation to the 3.9 Mb target region), for 1500 ng 54% and for 3000 ng 62%. This needs to be considered when the input amounts are reduced and when homozygous versus heterozygous gene loci are compared. In comparison to SNV callings, InDels do not follow the expected bimodal distribution for variant/reference ratios but resemble rather a Bernoulli distribution (Figure 2B). Based on these findings, we chose to discard InDels with variant/reference ratios lower than 15% from further analysis.

For the SNVs and InDel detection we next asked how reproducible they are and how high the coverage needs to be to minimize the error rates. Since all three preparations originated from the same tumor DNA and only the amount of input DNA differed, identical SNVs and InDels should be called. We therefore investigated if SNVs and InDels called for each amount of DNA were found in the other preparations with different amounts of DNA. With a minimum coverage of 3×, we found more than 98% concordance between two samples for SNVs (Figure 2C). Interestingly, when we looked at SNVs, which had been already annotated in the dbSNP database (referred to as 'known SNVs'), the concordance rates are even higher reaching about 99% at 3× coverage. In contrast, when we looked at SNVs which had not been annotated so far ('unknown SNVs'), concordance rates below 55fold coverages were up to 30% lower than for 'known' loci (Additional file 1, Figure S4). At coverage rates of 55× or more, SNV concordances were higher than 98% for 'known' and 'unknown' loci alike. For InDels we found concordance rates of 98% at above 20× coverage (Figure 2D), and we observed much smaller differences between known and unknown positions (Additional file 1, Figure S5).

### Distinct biopsies from a single tumor have identical somatic SNV profiles in selected prostate cancer candidate genes, but differ in their copy number patterns

A long-standing question of cancer research is whether biopsies are true representatives for the tissue of origin. This is of particular interest since many solid tumors grow as distinct tumor foci. We therefore addressed the questions whether biopsies from prostate tumors are uniform or if they are associated with different mutational patterns or different copy number variations. Prostate cancer is a prototype tumor to address this problem. The majority of these tumors are multifocal and in many cases two or more distinct, locally separated tumor foci can be identified [30,33].

We designed a target gene set of 1121 genes carefully selected by association to prostate cancer, cancer in general and signal transduction pathways. DNA was isolated from two different loci for each tumor in addition to matched benign tissue from frozen radical prostatectomy specimens of three prostate cancer patients. Selection of different foci was based on differences of histological and morphological phenotypes and was performed and controlled on the basis of HE stainings and P63/AMACR double immunostainings. The basal cell marker P63 decorates only benign glands whereas AMACR is a marker for tumor cells. In each case the two tumor foci analyzed displayed different histopathological gradings, in two cases Gleason patterns 3+4 in the low grade focus and 4+5 in the high grade focus, the third case displayed an additional tertiary pattern 5 in the high grade focus (See also Table 2 and Additional file 1, Methods).

**Table 2:** TMPRSS-ERG Fusion status and somatic mutations of the different foci analysed

patient-ID	Gleason Score	TMPRSS2-ERG Fusion	somatic substitutions
**Pat3_ normal**			
**Pat3_Focus 1**	4+5	Deletion	SH3BGR chr21:40883678
**Pat3_Focus 2**	3+4	no Fusion	

**Pat4_ normal**			
**Pat4_Focus 1**	3+4	no Fusion	SH3BGR chr21:40883671
**Pat4_Focus 2**	3+4(5)	no Fusion	

**Pat5_ normal**			
**Pat5_Focus 1**	3+4	no Fusion	NUB1 chr7:151053043
**Pat5_Focus 2**	4+5	Deletion and Insertion	

**Figure 3:**
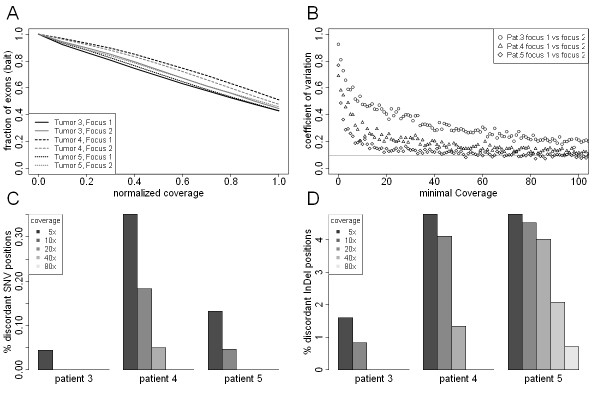
**Comparison of different tumor biopsies for targeted re-sequencing approaches**. (**A**) Normalized coverage-distribution plot for two foci of each of three tumor tissues. The mean coverage per exon was divided by the overall mean coverage of all exons and plotted as normalized coverage (x-axis). The fraction of bait-covered exons in the genome achieving coverages equal or lower than the overall mean coverage is indicated on the y-axis. (**B**) Exonwise coverage comparison of two foci of each of three tumor tissues. A coefficient of variation is calculated for each comparison and plotted by the smallest coverage of each exon-exon comparison. (**C**) Comparison of SNVs detected in two foci of each of three tumors. Discordant SNVs are those detected in focus A but not focus B and vice versa. (**D**) Comparison of InDels detected in two foci of each of three tumors. Discordant InDels are those detected in focus A but not focus B and vice versa.

Targeted enrichment with subsequent sequencing was performed with these nine tissue samples. We found the enrichment efficiency to be very similar for all samples: Approximately 69% of sequencing reads mapped to the target region and about 99% of targets were hit at least once (Table 1 and Additional file 2, Table S3). In addition, the coverage profiles were very similar for all patients as demonstrated by the cumulative normalized coverage plot and the coefficients of variation (Figure 3A, B).

For a comparison of the SNV profiles we used a two step procedure for loci covered in both preparations at a minimal coverage level of 20×. First, called SNVs for focus A were required to have at least 15% of reads containing the SNV. In the second step focus B was then analyzed and a SNV was considered concordant if the SNV was found in at least one read of focus B. Although the SNVs differed substantially between patients, we found no discordant position in any two foci of the same tumor in the three patients at this level of stringency. We also determined the concordance of SNV profiles at smaller coverage levels (Figure 3C). At a minimal coverage of 5× we observed 0.4% discordant loci at maximum but this difference is most likely caused by an amplification bias rather than by real differences, since the number of discordant foci quickly diminishes with rising coverage demands. We analyzed small InDels in a similar way and found again higher rates of discordance as compared to SNVs (Figure 3D). Except for one discordant locus found in Patient 5, no discordances were found when higher coverage cutoffs were used. We also investigated potential somatic SNVs by comparing each individual focus with its matched benign tissue. We found one somatic SNV for each of the three patients. This mutation was identified in both tumor foci but not in the benign tissue (Table 2).

**Figure 4:**
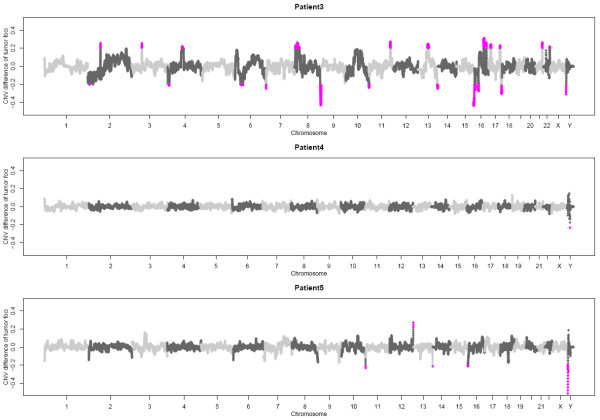
**Comparison of copy number profiles of different tumor biopsies**. DNA read frequencies and subsequent normalized log ratios for tumor versus normal were determined for chromosomal intervals (bins) of 55-190 Kb. Copy number changes were calculated as running median of the log ratios of 20 bins. Differences in copy number between the two foci of one tumor are depicted as the difference of the two running median vectors. Differences greater or equal 0.2 were highlighted in magenta.

In addition to the SNV profiles for the different tumor foci we also investigated the copy number variations within each focus. For this, we generated low-coverage whole genome sequencing profiles for the two tumor foci and the corresponding benign tissue from each patient. We determined potential somatic copy number variations by comparing each tumor focus with the matched benign tissue (Additional file 1, Figure S6). Received copy number variations were then compared between the two foci and the difference was plotted genome-wide (Figure 4). For patient 3 we found clear differences on chromosome 4, 10 and 13 between both foci with regard to CNVs (Additional file 1, Figure S6 A-C). In comparison, the biopsies taken from patient 4 and 5 seemed more homogeneous as no differences of the CNV profiles were apparent between the two tumor foci. Marked differences for patient 5 are located towards chromosome ends and visual inspection proposed the individual CNVs to be artefacts (Additional file 1, Figure S6 D).

## Discussion

Next-generation technologies such as targeted re-sequencing platforms are powerful tools for identifying genetic variations in cancer samples. Using prostate cancer as an example, we have assessed the use of different kinds and amounts of tissue samples for identifying genetic variations. In particular, we have investigated three aspects which are frequently addressed from oncologists and pathologists:

The first is whether or not it is possible to use FFPE material in addition to snap frozen material. The use of FFPE material would open up a large collection of tissue samples for molecular studies since most of the materials stored at pathology departments around the world are archived in this way. However, the preparation procedure of FFPE tissue with formaldehyde fixation and long-term storage at room temperature may generate DNA mutations and result in the identification of false SNVs or InDels. We previously showed that it is possible to use FFPE material for copy number analysis of whole genome data, although a higher sequencing capacity is required to achieve a comparable coverage [28]. Now we have extended our studies to targeted enrichment methods and found an uniform enrichment irrespective of the kind of tissue material used. Looking at the numbers of SNVs detected we found 0.98% false positive SNVs in FFPE preparations at a coverage level of 20× which can be strongly reduced at higher coverages (> 80×). Potential false positive SNVs can be explained by processes likely to occur during formalin fixation, like deamination and depurination processes. Our data suggests that the damage done by the FFPE preparation has a random distribution across all DNA fragments and can be corrected by sequencing depth. Since coverage levels of 80× and higher can easily be reached by targeted re-sequencing approaches, we recommend to use such high coverages when analyzing FFPE material. The same holds true for false negative SNVs. Keeping in mind that SNV detection is the main focus of DNA sequence analysis in cancer, the detection of small insertions and deletions becomes increasingly important. We therefore investigated if preparation of DNA from FFPE tissue may have an adverse effect on InDel detection. While the relative amount of discordant InDel positions is about 7 times higher than the amount of discordant SNV positions, we observed the same low discrepancy rates at higher coverage levels. Again, no discordance was found at a coverage level of 80×. Taken together, snap frozen tissues remain the preferred source of DNA, but FFPE tissue can be used for SNV and InDel detection instead if the coverage is increased. Furthermore, for certain clinically relevant questions, like for the detection of germline variants, e.g. when for a snap frozen tumor tissue no adequate matching benign tissue material is available, FFPE tissues can be used. In this case, the positive error rate obtained with FFPE material plays an inferior role.

The second methodological issue relates to the amount of material required. Decreasing the input amount of DNA to 500 ng still yielded good enrichment results, an even coverage and a highly reproducible calling of known genetic variants. However, we find increased redundant reads (reads with identical first positions) and a slightly higher variance of variant/reference ratios with decreased amounts of starting material. This suggests that - with these enrichment technologies - the minimal amount of input DNA cannot easily be reduced beyond 500 ng. Notably, the comparison among average and high amounts of DNA (1.5 μg vs 3 μg) performed better than a comparison including the lowest amount of DNA (500 ng).

While InDels detected show a variant/reference ratio distribution clearly deviating from the expected bimodal distribution and visible differences for the three DNA amounts, InDels are still highly reproducible above a coverage level of 45× for all amounts of DNA. We conclude that a decrease to 500 ng of input DNA is possible, but the benefit has to be weighed against the high coverage demands and potential challenges to SNV and InDel categorization.

The third challenge presented in our study consists of the heterogeneity of tumor tissue. In order to obtain results representative for the whole tumor, the amount and location of biopsies necessary is unknown. So far, it is not decided whether primary prostate cancers have a multifocal origin and thus are composed of multiple genetically distinct cancer cell clones or not. Currently, an independent clonal nature of multiple foci is considered since healthy men below 40 years frequently show presence of focal histological aberrations [34-36] many of which give rise only to latent prostate cancer, while clonal evolution of a few foci paves the way to clinically detectable disease [33,37-40]. On the other hand, prostate cancer metastases from different locations but from the same patient show a surprisingly similar pattern with regard to copy number alterations [41-43]. Experiments available to address this question include the determination of the DNA ploidy, micro-satellite analysis, c-myc amplifications with FISH, DNA methylation or the TMPRSS2-ERG fusion status on separate tumors within the same prostate [44]. In our hands, using samples derived from different foci within one prostate tumor and performing DNA re-sequencings of prostate cancer relevant genes, we found almost identical distributions of mutations within different foci of the same patient. Notably, SNV profile concordance was 100% for all three patients at coverage levels above 20×. Even tumor parts with different TMPRSS2-ERG gene fusion status are remarkably identical with regard to small nuclear variations. In addition, focusing on somatic mutations, we find no differences between different tumor foci. However, although we focused on prostate cancer candidate genes, the low number of somatic mutations in prostate cancer and the fact that we only analyzed ~10% of the exome prohibit a generalized conclusion. Recent studies, such as Taylor et al with 0.31, Kan et al with 0.33, and Berger et al with 0.9 non-synonymous mutations per Mb, suggest low somatic mutation rates per Mb for prostate cancer [8,9,45]. In line with this somatic mutation frequency we found only one somatic mutation for each of the three patients. The sensitivity of current re-sequencing approaches might further explain the missing focal diversity. Irrespective of the low frequency of somatic mutations we detected in the tumor samples we found large aberrations in copy number. We have used a whole genome re-sequencing approach to detect somatic copy number variations for each focus and compared the two foci from the same tumor. Interestingly, for one patient with clear differences in the TMPRSS2-ERG fusion pattern, we also find significant differences between the two foci, whereas for two other patients no significant CNVs can be detected. Along this line Navin et al. used a modified comparative genomic hybridization (CGH) technology to study the clonal composition of breast tumors and found a large proportion of monogenomic tumors and only a small fraction of tumors with a heterogenomic foci structure [46]. Our results would implicate that the location of biopsies taken within tumors is of minor relevance for the detection of mutations, but plays a major role for the detection of copy number variations. Within this direction, recent publications also suggest that genomic rearrangements are a major genetic factor underlying prostate cancer [47]. Since we did not perform 3D reconstructions of the whole tumors our approach cannot be used to answer the question of multifocal origin of heterogeneous prostate tumors. Even for the estimation of tumor heterogeneity our studies are most likely an underestimation, because we are investigating tissue samples with a complex composition of single cells. Thus, the genetic profiles are the sums over all cells contained within the section and might mask the true tumor heterogeneity. At the moment we are extending our analysis onto a single cell level to further gain insight into the evolutionary architecture of prostate tumors. With this we might be able to pin down the true tumor composition and we might even identify tumor stem cells on a genetic level. However, since we find differences between different biopsies from the same tumor on a copy number level, we can conclude that several biopsies need to be investigated to gain insight into the genomic context of prostate cancers based the overall tumor heterogeneity.

Furthermore, with the technologies described we are now in the progress to extend our analyses to large sample cohorts from pathology departments where we can select tissue specimens from specific clinical studies. This enables us to address clinical relevant questions such as progression and therapy resistance of tumors which is an important step towards the application of targeted re-sequencing approaches as routine diagnostic tools in oncology.

## Conclusion

Illumina sequencing is a powerful tool for large-scale re-sequencing projects. For clinical applications, in particular for the benefit of cancer patients, several key issues need to be addressed: Tissue material, input amounts and reproducibility of the data in regard to tumor heterogeneity. Our optimized protocols guide through each of these issues and provide data for an optimal strategy for the usage in clinical settings. We show that FFPE material can be used with higher coverages as substitution of cryo-frozen tissue and that it is in particular useful for the determination of germline variations when tumor tissues have already been sequenced. Lowering the amount of input material results in an increase of redundant reads and a slightly higher variance of variant/reference ratios, but can be overcome to a certain degree with adequate analysis tools. Finally, the tumor heterogeneity plays an important role for the detection of copy number variations, but is of minor importance for the detection of somatic variations. This implies that the sampling of tumor tissues is of major importance and needs to be taken into consideration for clinical diagnostic purposes.

## Competing interests

The authors declare that they have no competing interests.

## Authors' contributions

Clinical samples and discussions were provided by GS, IV, GB and HK. MK, MI, BT and MRS carried out the experiments, data analyses were done by MK, MI, HK and MRS; HS and MRS conceived and coordinated the project; MK, MI, HS, RH, SK, HK, HL and MRS wrote the paper. All authors read and approved the final version.

## Pre-publication history

The pre-publication history for this paper can be accessed here:

http://www.biomedcentral.com/1755-8794/4/68/prepub

## Supplementary Material

Additional file 1 **Supplementary Methods, Figure Legends S1-S6, Table Legends S1-S3**. Figures S1-S6 Click here for file

Additional file 2 **Tables S1-S3**. Click here for file
