# Supplementary material for: Targeted high throughput sequencing in clinical cancer Settings: formaldehyde fixed-paraffin embedded (FFPE) tumor tissues, input amount and tumor heterogeneity
Source: BMC Med Genomics. 2011 Sep 29;4:68. doi: 10.1186/1755-8794-4-68 (PMC3192667; doi:10.1186/1755-8794-4-68)
Supplement: Additional file 1 — Supplementary Methods, Figure Legends S1-S6, Table Legends S1-S3. Figures S1-S6 [file 1755-8794-4-68-S1.PDF]

Supplementary Information for Kerick *et al.*, Targeted High Throughput Sequencing in Clinical Cancer Settings: Formaldehyde fixed-paraffin embedded (FFPE) tumor tissues, input amount and tumor heterogeneity.

I. Supplementary Methods

II. Supplementary Figures and Table Legends

III. Supplementary Figures

I. Supplementary Methods

Selection of prostate cancer cases

Selection of different foci was based on differences of histological and morphological phenotypes and was performed and controlled on the basis of HE stainings and P63/AMACR double immunostainings. P63 as a basal epithelial cell marker is absent in tumors, and tumor cells are positive for AMACR. In each case the two markers displayed different histopathological gradings, in two cases Gleason patterns 3+4 in the low grade focus and 4+5 in the high grade focus, the third case displayed an additional tertiary pattern 5 in the high grade focus (Table 1).

Table 1: Histopathological characteristics:

Patient	Whole Tumor Histopathology	Low grade focus Histopathology	High grade focus Histopathology
Pat 3	GS 9 (4+5)	GS 7 (3+4)	GS 9 (4+5)
Pat 4	GS 7 (3+4)	GS 7 (3+4)	GS 7 (3+4), tert.pattern 5
Pat 5	GS 7 (3+4)	GS 7 (3+4)	GS 9 (4+5)

Asservation and workup of radical prostatectomy specimens

In order to ensure high quality tissue samples for molecular biological analyses we adopted a workup procedure that minimized exposure to ischemic conditions. For that the surgeon performing a radical prostatectomy interupts blood flow to the prostate as late as possible. The specimen is announced to the pathologist 15 – 20 min before the specimens is removed and the technician prepares everything for a rapid work-up procedure. After removal the prostate is placed into two sterile gloves and is sent in a tubing transport system to the pathology lab where workup is started immediately in a sterile flow working bench. The specimen is first weighed and the surface is treated with black ink to mark the margins. Next a transversal, 4 mm thick slice of the prostate specimen is cut out, divided into for parts, embedded in Tissue-Tek OCT Compound and rapidly frozen in liquid nitrogen for asservation of frozen tissue. This step is usually finished about 10 min after the prostate had been removed from the patient. The rest of the prostate is then fixed in buffered formalin solution for 24 hours. The next day the fixed tissue is sectioned and cut into tissue blocks which are then dehydrated and embedded in paraffin according to the standard pathological procedure. All tissue pieces of the specimen are collected and annotated with regard to their position and orientation in the specimen. HE sections are prepared from all paraffin and frozen tissue blocks for histological classification of the tumor. When all examinations for pathological diagnosis are finished the tissue samples can be used for research analyses. The paraffin blocks are stored at room temperature, frozen tissue blocks at -80° C.

DNA isolation from paraffin embedded tissue.

A 3 µm section of the paraffin blocks was prepared and HE stained. The pathologist identified the tumor areas and selected and marked the tumor areas and the benign control areas to be isolated for DNA preparation. The areas of interest were then copied from the HE section to a transparent film and from there to the tissue block using a needle (Fig. 1). The border of the areas to be isolated were caved with a cutter and subsequently 10 µm sections were cut from the blocks. Upon cutting the benign and cancer areas of interest fell out of the sections and were collected with a pipette tip and transferred into DNase/RNase free 1.7 ml

microcentrifuge tubes (Eppendorf). Several sections were used to obtain altogether about 5-10 cm² of tissue section per sample.

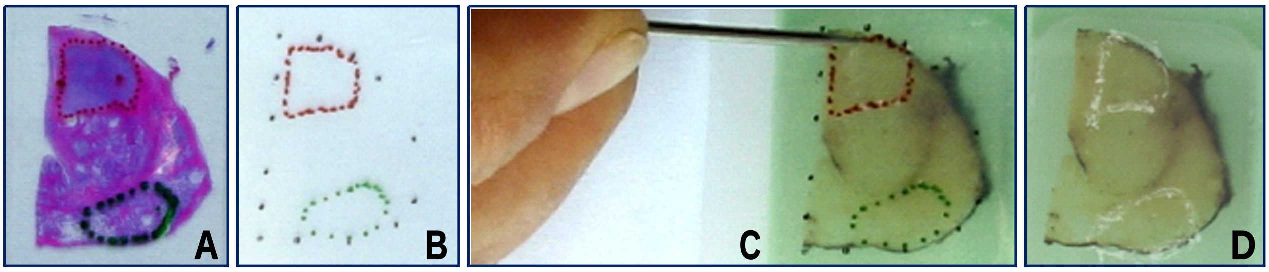

Figure 1 A *- Tumor and benign control region are identified and marked by the pathologist (red = tumor, green = benign) on a HE stained section of the tissue block.* ***B*** *–Marked regions are copied to a laboratory membrane and* ***C*** *– transferred to the tissue block using a needle sticher.* ***D*** *– the border of the areas is carved with the needle before the sections for DNA isolation are cut.*

II. Supplementary Figures and Tables Legends

Supplementary Figure S1. Comparison of FFPE and snap frozen tissue material for targeted whole exome (A, B) and custom design 3.9Mb targeted re-sequencing (C-F). (**A**) Cumulative coverage plot for the snap-frozen and FFPE embedded tissue. Shown are the normalized coverages for the corresponding fractions of exons (baits). (**B**) Sorted coverage plot for the snap-frozen and FFPE embedded tissue. The y-axis depicts the coverage (log10 scale), while the x-axis depicts all exons examined sorted according to their coverage. (**C**) Exonwise coverage comparison of snap frozen and FFPE DNA preparations for tumor and benign tissue samples. Coefficient of variations are calculated for each comparison and plotted by the smallest coverage of each exon-exon comparison. (**D**) Mean coverage by GC content for snap frozen and FFPE DNA preparations. All exons were split into 800 bins by GC content and the average exon coverage was averaged within each bin. (**E**) Comparison of SNVs detected in snap frozen and FFPE DNA preparations for tumor and benign tissue samples. False negative SNVs are detected in the snap frozen preparation but not in the FFPE preparation. False positive SNVs are detected in the FFPE preparation but not in the snap frozen preparation. (**F**) Comparison of InDels detected in snap frozen and FFPE DNA preparations for tumor and benign tissue samples. False negative InDels are detected in the snap frozen preparation but not in the FFPE preparation. False positive InDels are detected in the FFPE preparation but not in the snap frozen preparation.

Supplementary Figure S2. (**A**) Cumulative coverage plot for different amounts of input DNA. Shown are the normalized coverages for the corresponding fractions of exons (baits). (**B**) Sorted coverage plot for different amounts of input DNA. The y-axis depicts the coverage (log10 scale), while the x-axis depicts all exons examined according to their coverage.

Supplementary Figure S3. (**A**) Cumulative coverage plot for two foci of each of three tumor tissues. Shown are the normalized coverages for the corresponding fractions of exons (baits). (**B**) Sorted coverage plot for two foci of each of three tumor tissues. The y-axis depicts the coverage (log10 scale), while the x-axis depicts all exons examined according to their coverage.

Supplementary Figure S4. Comparison of known (black) and unknown (blue) SNVs detected with different amounts of input DNA; separation between in dbSNP annotated (‘known’) and not annotated (‘unknown’) locations.

Supplementary Figure S5. Comparison of known and unknown InDels detected with different amounts of input DNA; separation between in dbSNP annotated (‘known’, black) and not annotated (‘unknown’, blue) locations.

Supplementary Figures S6. Comparison of copy number profiles of different tumor biopsies. Exemplarily we depicted the CNV profiles of three chromosomes from patient 1 (**A-C**) to demonstrate CNV differences between the tumor foci and one profile of patient 3 (**D**) to demonstrate the artefacts found at the chromosomal ends. DNA read frequencies and subsequent normalized log ratios for tumor versus normal were determined for chromosomal intervals (bins) of 55-190 Kb. Copy number changes were calculated as running median of the log ratios of 20 bins. Differences in copy number

between the two foci of one tumor are depicted in a separate subplot as the difference of the two running median vectors. Differences greater equal 0.2 were highlighted in magenta.

Supplementary Table S1 – Genomic regions for the custom-designed targeted re-sequencing (3,9Mb approach)

Supplementary Table S2 – Sequencing statistics

Supplementary Table S3 – Enrichment statistics

Supplementary Figure S1

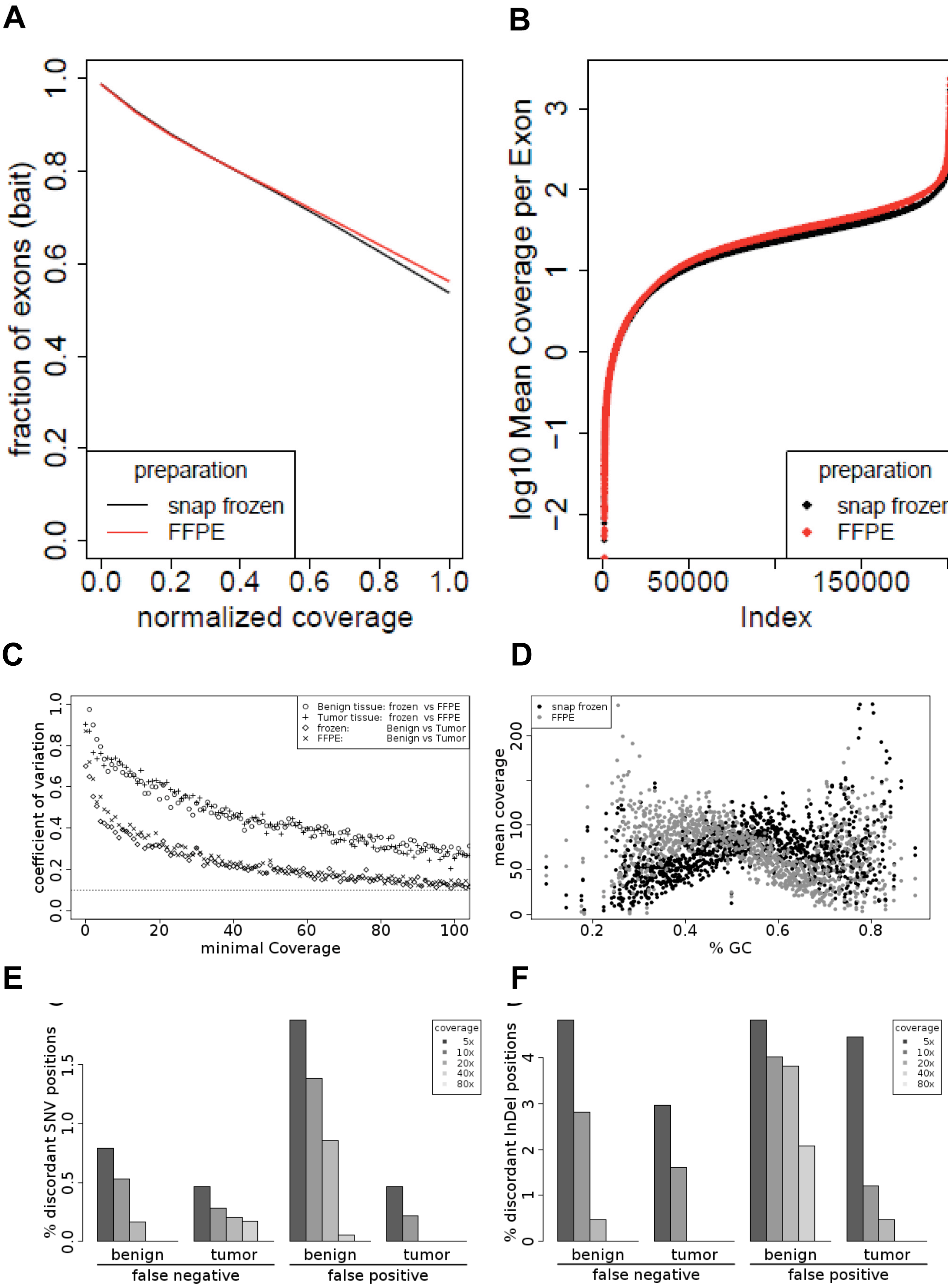

Supplementary Figure S2

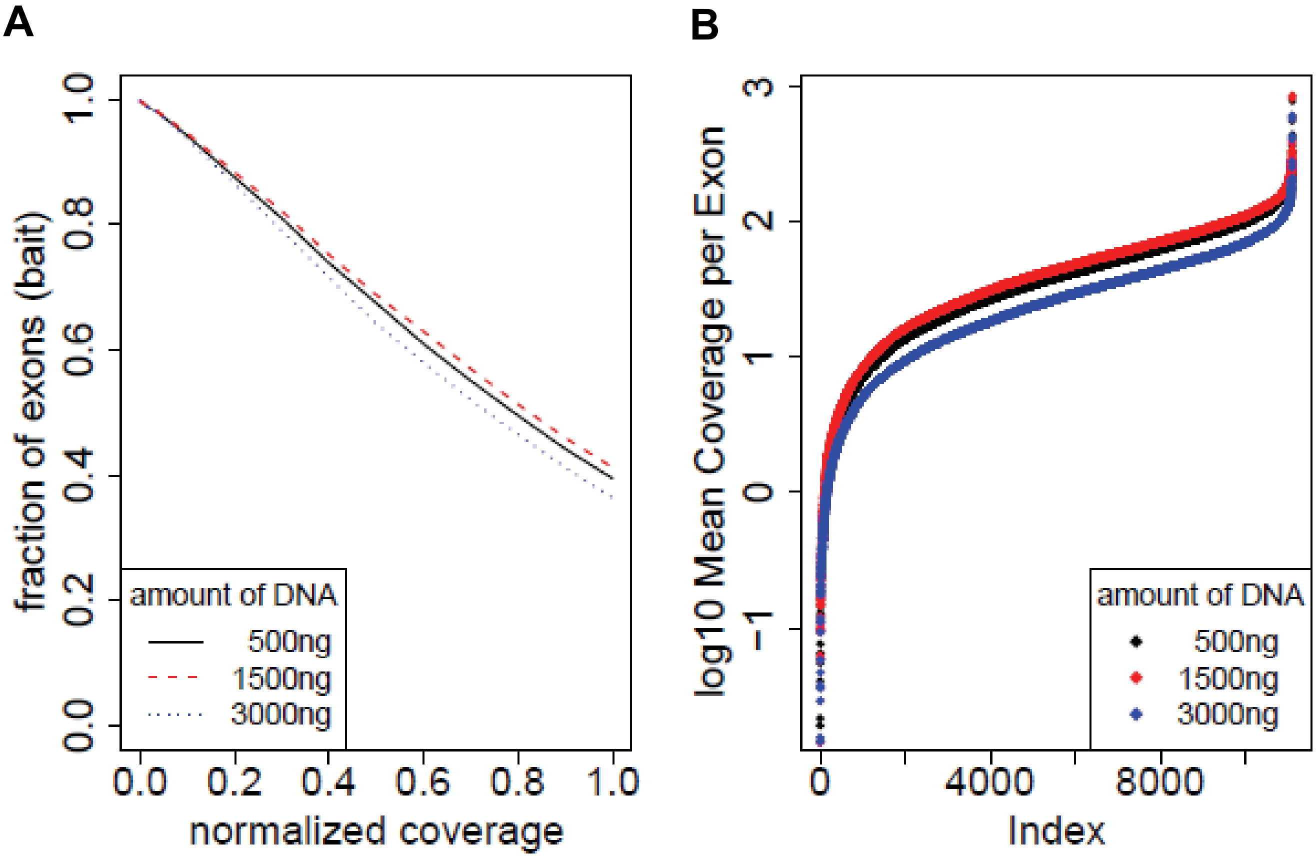

Supplementary Figure S3

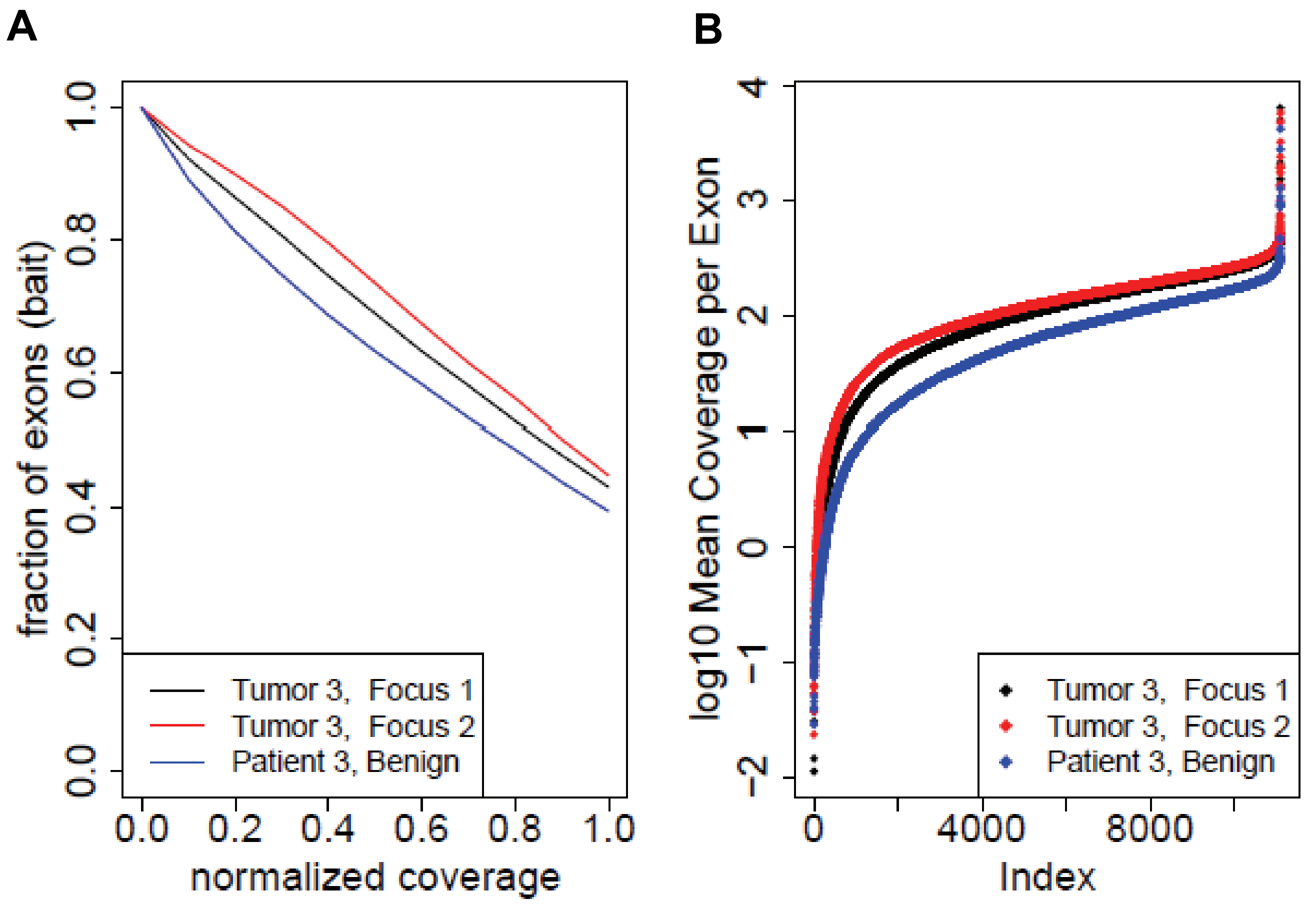

Supplementary Figure S4

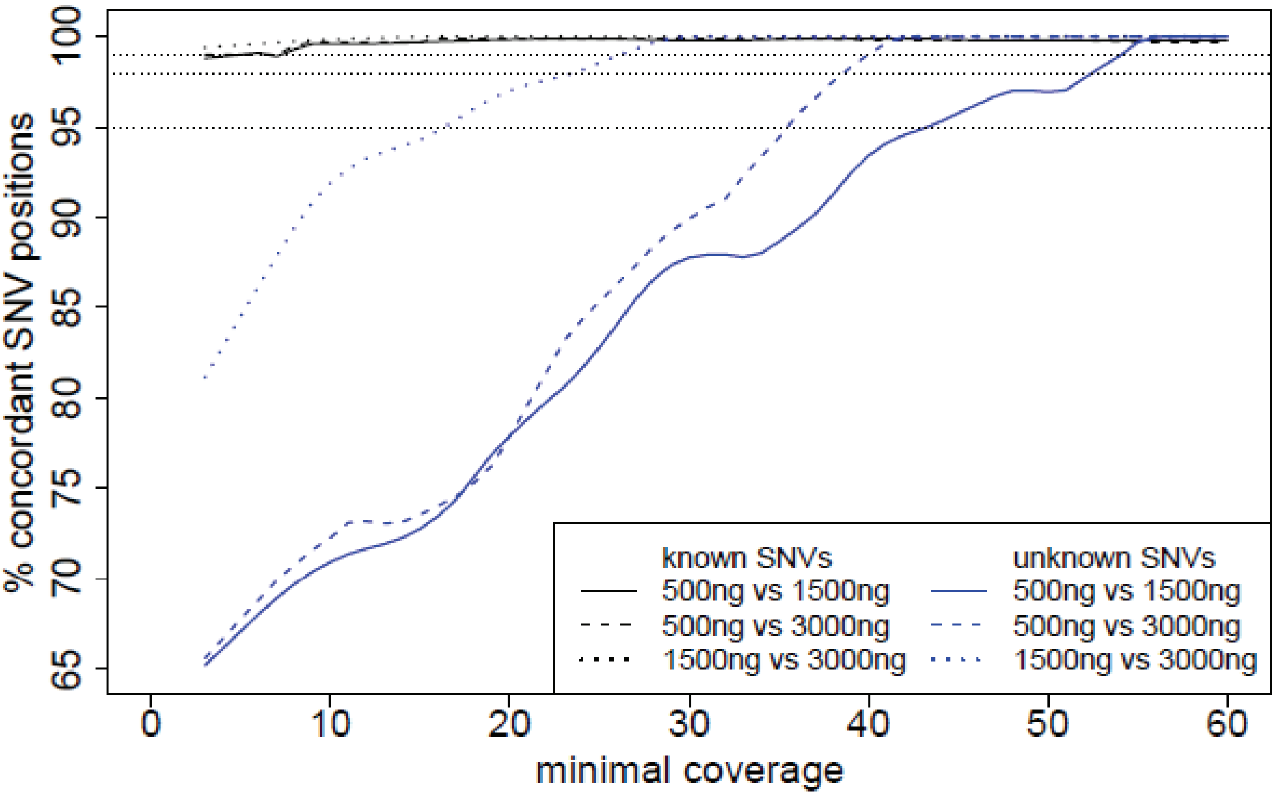

Supplementary Figure S5

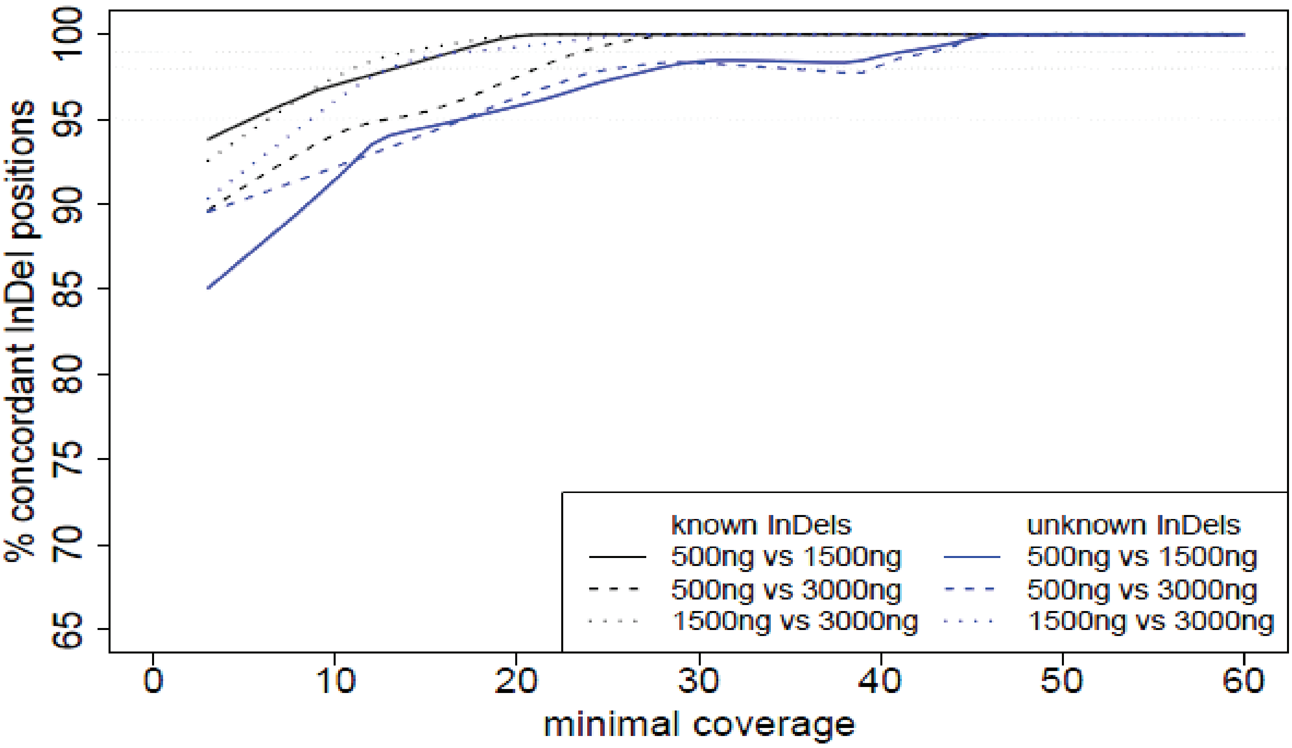

Supplementary Figure S6

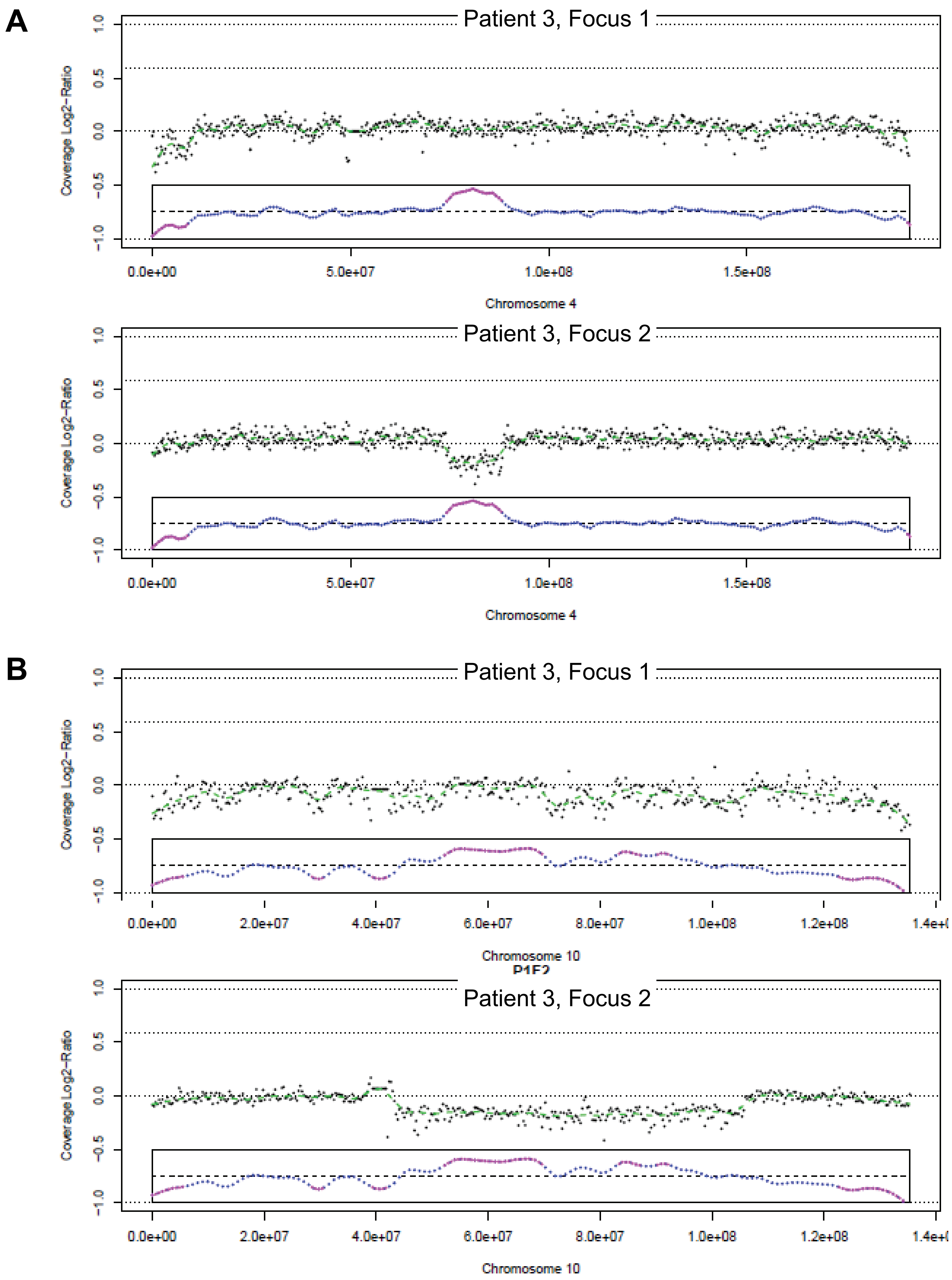

C

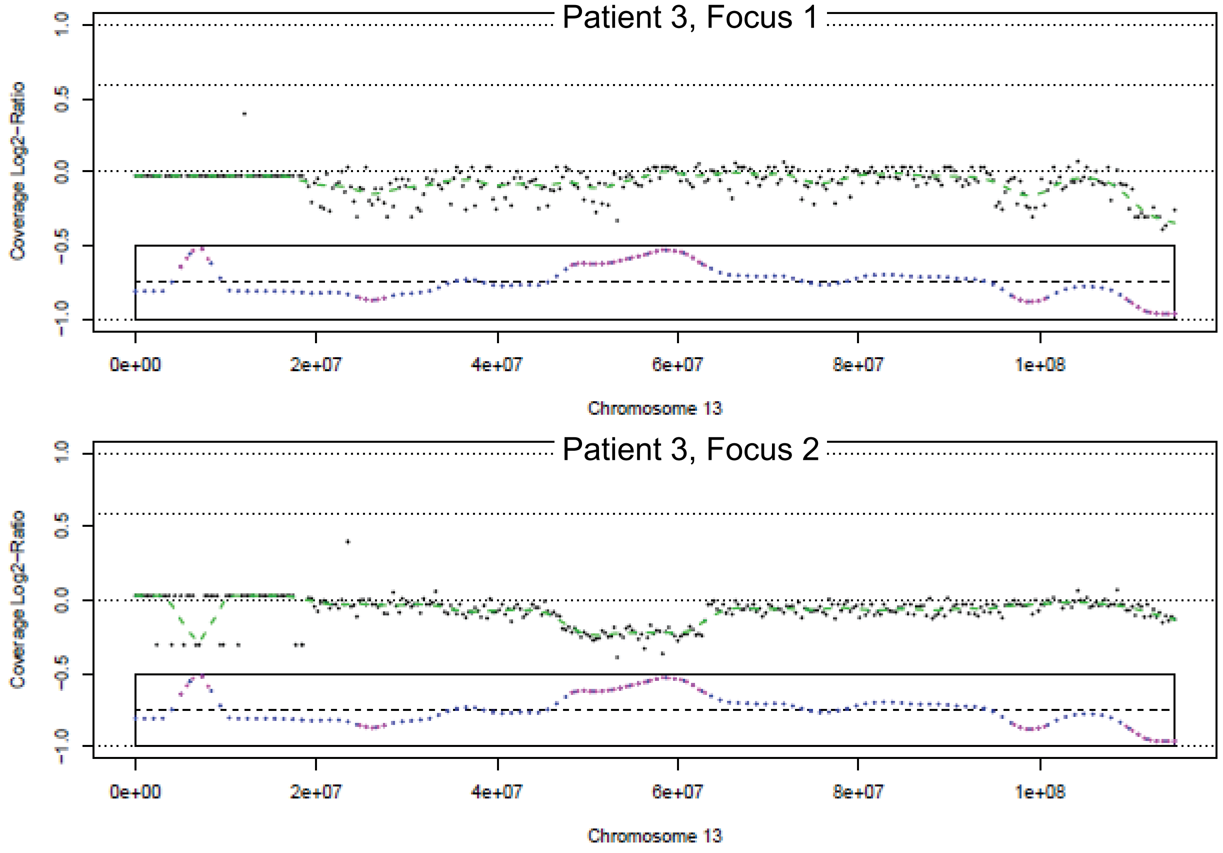
Patient 3, Focus 1
Coverage Log2−Ratio
Chromosome 13
Patient 3, Focus 2
Coverage Log2−Ratio
Chromosome 13

D

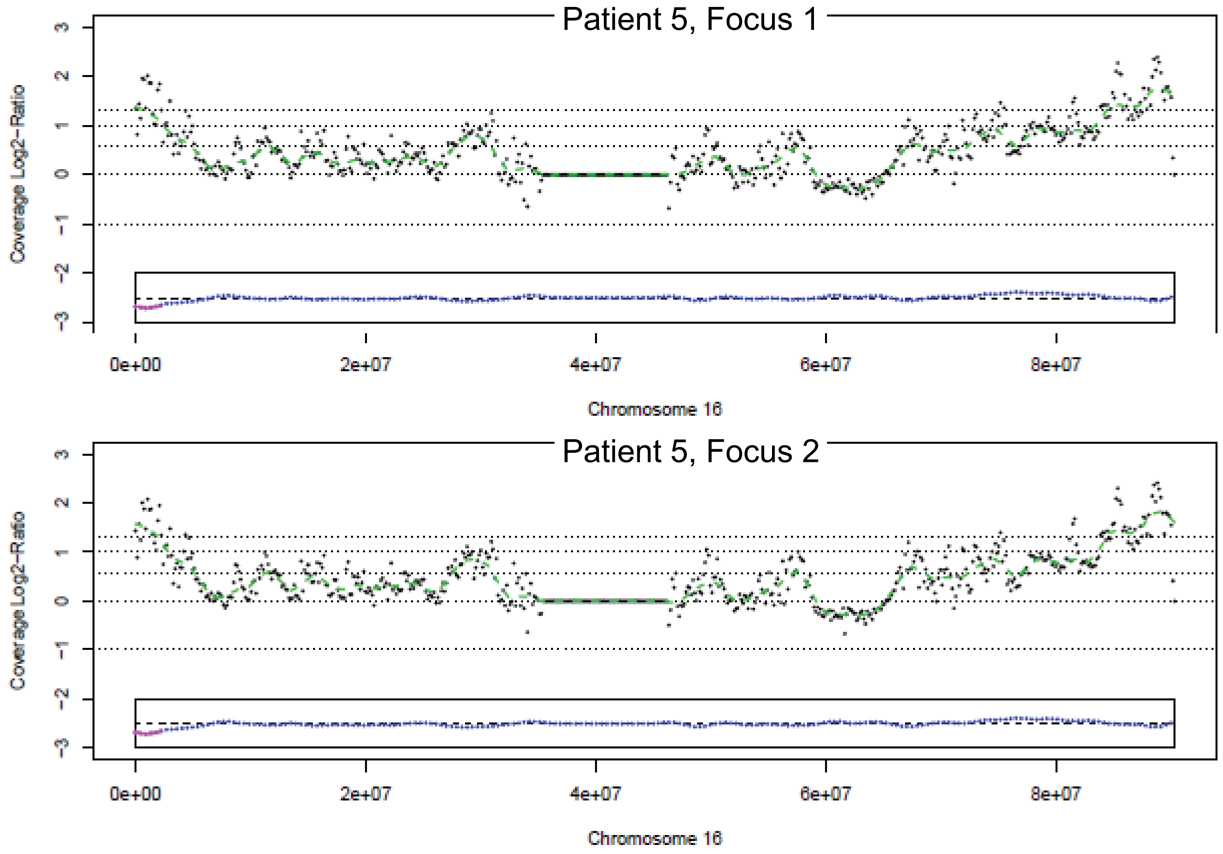
Patient 5, Focus 1
Coverage Log2−Ratio
Chromosome 16
Patient 5, Focus 2
Coverage Log2−Ratio
Chromosome 16
